# NDVI-derived forest area change and its driving factors in China

**DOI:** 10.1371/journal.pone.0205885

**Published:** 2018-10-17

**Authors:** Lizhuang Liang, Feng Chen, Lei Shi, Shukui Niu

**Affiliations:** 1 College of Forestry, Beijing Forestry University, Beijing, China; 2 State Forestry Administration Key Laboratory for Science & Technology of Bamboo & Rattan, International Center for Bamboo and Rattan, Beijing, China; Universidade Federal de Mato Grosso, BRAZIL

## Abstract

China harbors diversified forest types, from tropical rainforest to boreal coniferous forest, and has implemented large-scale reforestation/afforestation programs over the past several decades. However, little information is available on changes in China’s forest area and the causes. In this study, we used the classified forest distribution thematic map derived from Normalized Difference Vegetation Index (NDVI) datasets and a revised IPAT model to examine China’s forest area change and the possible driving factors from 1982 to 2006. Overall, NDVI-derived forest areas were numerically consistent with those reported in the 3^rd^, 4^th^, 5^th^, and 6^th^ National Forest Inventories, respectively. Over the past 25 years, China’s forest area was estimated to have an average of 169.18 million hectares with an annual increase of 0.15 million hectares (*c*.*a*. a total net increment of 3.60 million hectares), which is equivalent to 0.089% of the relative annual change rate. However, a large difference in the changing rate and direction of forest area at the province level was found; for instance, forest area has declined in 10 provinces, mainly in Northeastern and Southern China, while 21 provinces showed an increase. The changes were most likely attributed to the policy regarding the import and export of timber and affluence (per capita gross domestic product), and both contributed more than 80% of the total contribution of the six factors of the revised IPAT model.

## Introduction

Forests covers 30.6% of the Earth’s landmass, and its change has a large effect on biodiversity, clean air and water, carbon emissions, etc. [1]. However, forest change is always underway in some region of the world [2, 3]. Thus, a better understanding of forest cover changes is urgently needed for researchers, forest managers and decision-makers.

China harbors diversified forest types, from tropical rainforest to boreal coniferous forest, and has implemented large-scale reforestation/afforestation programs over the past several decades [4]. According to the 8^th^ National Forest Inventory (NFI), China has a forest area of 207.69 million hectares [5], accounting for approximately 5.15% of the global forest area. A comprehensive assessment of China’s forest cover change and its driving factors is important for clarifying the nature of regional and global forest change.

Generally, forest cover change can be monitored via NFI [6] and/or remote sensing (RS) data [7, 8]. However, Chinese NFI data lack spatial georeferenced records and can not be updated in a timely manner. In contrast, two-decadal advancements in remote sensing (RS) techniques and developed vegetation indexes make the real-time monitoring of forest change and its causes possible [9–11]. For instance, Janssen et al. [12] detected forest cover change in a nature reserve of central Ghana using the Normalized Difference Vegetation Index (NDVI). Li et al. [13] used NDVI to examine land cover change in Hangzhou Bay. Zhao et al. [8] used NDVI to explore long-term vegetation changes and their drivers on the Mongolian Plateau. It should be noted, however, that the inconsistency was frequently found in forest cover change based on NFI and RS data, especially on a large scale [14–16]; for instance, the Chinese 8^th^ NFI (2009–2013) indicated an increasing forest area compared to the 6^th^ NFI (1999–2003), but the Global Forest Change dataset [17] showed a net loss of almost 40,000 km^2^ during the 2000s (2000–2012).

Currently, there are three more widely used NDVIs derived from Landsat TM/ETM+, Moderate-resolution Imaging Spectroradiometer (MODIS) or NOAA Advanced Very High Resolution Radiometer (AVHRR) imagery. Landsat imagery has relatively high spatial-temporal resolutions but a low temporal frequency of acquisition because of cloud masking, and finding cloud-free images to cover all of China may require imagery from several different years or a combination of data from different satellites. Landsat NDVI may result in inconsistent time and biased results because of inconsistent data sources while exploring the forest cover change and its driving factors. The MODIS NDVI dataset has only been available since 2001, thus limiting the time span that can be studied. AVHRR imagery has a coarse spatial resolution but a high temporal frequency that does provide global data, and NDVI datasets developed from the Global Inventory Monitoring and Modeling Studies Working Group (GIMMS) [18–20] are thus more appropriate and widely used in large-scale mapping and vegetation cover change.

In this study, GIMMS NDVI datasets were used to examine China’s forest area change and its driving factors. Specifically, we intended to examine (1) whether NDVI-derived change in forest area is consisted with NFI; (2) how China’s forest area has changed over the past 25 years; and (3) what the possible driving factors were.

## Data and methods

### Data

#### Vegetation Map of China

A Vegetation Map of China at 1:1,000,000 was plotted by primarily using ground survey data from the 1980s and recorded 175 forest types (please see S1 Fig) [21]. It contains almost all types of forests in the world, from tropical rain forest to boreal coniferous forest, covering tropical, subtropical, temperate and cold temperate climatic zones. For more details, please see http://www.nsii.org.cn/mapvege.

These data provide one of the auxiliary maps for land cover classification during the process of interpretation and the basis for the subdivision of China’s forest types (please see the later Section Methods).

#### NDVI datasets

NDVI datasets was downloaded from the Global Inventory Monitoring and Modeling Studies Working Group [18–20] with a time span from 1982 to 2006 and with spatial and temporal resolutions of 8 km and 15 days, respectively. These datasets have been widely used to evaluate vegetation changes and can be downloaded at http://iridl.ldeo.columbia.edu/SOURCES/.UMD/.GLCF/.GIMMS/.NDVIg/.global/.dataset_documentation.html.

NDVI datasets are most likely influenced by extreme climate in one certain year [22]. To eliminate the adverse effects induced by extreme climate, multi-year NDVI datasets were composed as a time period. In addition, to utilize the Sixth National Forest Inventory Distribution Map as an auxiliary map for visual interpretation, and make a comparison of forest area derived from NDVI and that reported by the 3^rd^, 4^th^ and 5^th^ NFI, NDVI datasets were grouped into six time periods, namely, 1982–1983, 1984–1988, 1989–1993, 1994–1998, 1999–2003 and 2004–2006.

There were 24 NDVI gridded images in any one calendar year. For each time period, all of the same half-month NDVI images in several years (2, 3 or 5 years) were respectively averaged, and then synthesized as a synthetical layer with 24 channels; the minimum, maximum and mean value of each pixel for each period can thus be extracted and reserved for the following expert classification of forest types. For more details of preprocessing NDVI datasets, please see the paper by Shi [23].

#### National Forest Inventory (NFI)

China has implemented eight National Forest Inventories since the 1970s, namely, 1973–1976, 1977–1981, 1984–1988, 1989–1993, 1994–1998, 1999–2003, 2004–2008 and 2009–2013 [20]. To date, a total of 415,000 permanent and temporary plots were set up in the country, and information on species composition, tree height, diameter at breast height and other relevant parameters for each plot was documented. Four NFIs were used in the current study (i.e., 1984–1988, 1989–1993, 1994–1998, and 1999–2003) [24–27]. The statistical data of forest resources for each province can be browsed at http://www.cfsdc.org/.

#### Auxiliary data for interpretation

During the interpretation, we also used the following auxiliary data:

The Sixth National Forest Inventory Distribution Map at a scale of 1:4,000,000 includes eight land use types, namely, coniferous forest, broadleaved forest, mixed forests, bamboo forest, shrub, water, desert and others (please see S2 Fig) [28].

Terrain data was downloaded from U.S. Geological Survey with a resolution of 1 km (https://lta.cr.usgs.gov/GTOPO30). It is first projected to the Albers Equal Area Projection, and then resampled to a resolution of 8 km for utilization in expert classification.

The MODIS Land Cover Type product (MCD12Q1, downloaded from https://lpdaac.usgs.gov/dataset_discovery/modis/modis_products_table/mcd12q1) consists of the 17-class International Geosphere–Biosphere Programme classification (IGBP), the 14-class University of Maryland classification (UMD), a 10-class system used by the MODIS LAI/FPAR algorithm, an 8-Biome classification and a 12-Class plant functional type classification [29, 30]. The product has a spatial resolution of 500 m and was completed in 2001; the completion time coincided with the 6^th^ NFI (Hereafter called IGBP 2001 in this paper). Given more land cover types (17 classes), the IGBP 2001 classification product was identified as a reference map for assessment of our forest cover interpretation accuracy.

#### Other data

The other data we used in the revised IPAT model are as follows:

Population and Gross Domestic Product (GDP) data were based on the previous statistical yearbooks and downloaded from the CEInet Statistics Database [31]. It is noted that China’s historical population data do not include Chongqing City and Sichuan Province, owing to the incompleteness of the records, and Hong Kong, Macao and Taiwan are also excluded.

National wood production data were compiled from the China Forestry Statistical Yearbook [32].

Data on wood imports was compiled from the China Foreign Economic and Trade Yearbook and Development Research Center of the State Council of China [33, 34].

Afforestation data at the provincial and national levels were compiled from “New China’s 50 Years of Agricultural statistics” [35] and “Compilation of Agricultural Statistics for 30 Years of Reform & Opening up” [36].

### Methods

#### Subdivision of China’s forest types

Based on the Vegetation Map of China, together with the climatic zone (tropical, subtropical, temperate and cold temperate) and life types (evergreen vs. deciduous, coniferous vs. broadleaved types), China’s forests were subdivided into 16 land cover types in this paper, namely, cold temperature and temperature deciduous coniferous forest, cold temperature and temperature evergreen coniferous forest, temperature evergreen coniferous forest, subtropical and tropical evergreen coniferous forest, temperature evergreen coniferous and deciduous broadleaved mixed forest, subtropical evergreen coniferous and evergreen broadleaved mixed forest, temperature deciduous broadleaved forest, subtropical deciduous broadleaved forest, subtropical evergreen broadleaved and deciduous broadleaved mixed forest, subtropical evergreen broadleaved forest, tropical rainforest, subtropical and tropical deciduous coniferous forest, shrub, bamboo forest, other vegetation and unvegetated types (for more details, please see the S1 Table).

#### NDVI-derived interpreted thematic map using expert classification method

An expert classification method is defined as land use classification using an expert system through supervised data training or subjectively defined by human experts and is widely used in large-scale vegetation classification [37–39]. This method requires the establishment of a set of decision trees (rules) in advance. Owing to the obvious discrepancy of NDVI values with land cover types and altitude, NDVI was frequently employed in decision trees [40–42]. In this paper, the minimum, maximum and mean NDVIs of gridded cells, together with a digital elevation model (DEM), was used while constructing the decision tree.

The Sixth National Forest Inventory Distribution Map was based on the results of the 6^th^ NFI. While drawing NDVI profiles and identifying the threshold of each forest type in building decision (or classification) trees by province, the Sixth National Forest Inventory Distribution Map, together with the Vegetation Map of China, were used as auxiliary maps. In other words, the decision tree was mainly based on the time period of 1999–2003. S3–S6 Figs show the interpreted forest distribution maps of the four time periods of 1984–1988, 1989–1993, 1994–1998 and 1999–2003 using an expert classification method.

In the accuracy assessment, IGBP 2001, at a spatial resolution of 500 m, was treated as a standard map. Before the accuracy assessment, the vegetation types of IGBP 2001 were subdivided to match the interpreted results following the climatic zones, the Sixth National Forest Inventory Distribution Map and the Vegetation Map of China. Please see S2 Table for more details of the subdivision of IGBP 2001.

Due to the large difference in the areas of various vegetation types, a hierarchical random point generation method was adopted to generate random points, whose total areas were not less than 25% of the area of each land cover types. S3 Table showed that the overall interpretation accuracy was high (84.18%), and the kappa was 0.82.

#### Land use transfer matrix

To quantify the mutual transformation between forest and non-forest, land use transfer matrix was used to explore the land-use transformations between 1982–1983 and 2004–2006 aided by the software, ERDAS Imagine (Leica Geosystems GIS & Mapping LLC, Atlanta, US). The transfer matrix reflects the change information of a specific location in a certain period. We can use the transfer matrix to calculate the decreased and increased area and the changing magnitude of each land use type. The formula for the land use transfer matrix can be written as:

$$S_{ij}=\left[\begin{array}{llll} s_{11} & s_{12} & \cdots & s_{1n}\\ s_{21} & s_{22} & \cdots & s_{2n}\\ \vdots & \vdots & \vdots & \vdots \\ s_{n1} & s_{n2} & \cdots & s_{nn} \end{array}\right]$$ (1)

where *S* represents the area, *i* and *j* are the land use types before and after the transformation, respectively, and *n* denotes the number of transferred land cover types.

#### Revised IPAT model for exploration of factors driving forest area change

Ehrlich and Holdren [43, 44] assumed that the human impact on the environment (*Influence*) results from the population (*Population*), its affluence (*Affluence*) and technological innovation (*Technology*) and can be expressed using the equation: *Influence* = *Population* × *Affluence* × *Technology*. Hereafter, this is referred to as the IPAT model (or equation). The equation is a very useful tool to dissect interactions and mutual influence and has been widely used to analyze the impact of human activities on the environment [45–47].

Some studies have indicated that forest cover dynamics are often driven by the population [48], economic development [49, 50], and the forestry policy [51, 52]. In this paper, we therefore assume that changes in forest area (*I*) are influenced by the population (*P*), level of economic development (*A*), technology (i.e., consumption intensity) (*C*), policy of importing wood (*T*), sustainable management level (*S*) and reforestation projects (*R*) and can thus be expressed using an equation that resembles the IPAT equation as follows: *I* = *P* × *A* × *C* × *T* × *S* × *R*, where *P* denotes the Chinese population, *A* denotes the per capita GDP, *C* denotes the total wood consumption intensity (i.e., the sum of wood imports and domestic wood) per unit GDP, *T* denotes the forestry policy on wood orientation (as indicated by the ratio of national forest-derived wood to the total amount of wood, a reflection of the wood-oriented policies), *S* denotes the sustainable forest management level (as indicated by the ratio of reforestation area to the domestic wood production) and *R* denotes the impact of afforestation on forest area change (as indicated by the ratio of forest change area to afforestation area). Table 1 shows more details on the symbols used and their implications.

**Table 1:** Symbols for the impact of changes in forest area and forces that affect them.

Category	Symbol	Dimension	Relative annual change rate in forest area (%)
Influence	*I*	Area	*i*
Population	*P*	Capital	*p*
Affluence	*A*	GDP/Capital	*a*
Wood consumption intensity	*C*	Total wood/GDP	*c*
Policy of importing wood	*T*	Domestic wood/Total wood	*t*
Sustainable management level	*S*	Reforestated area/Domestic wood	*s*
Impact of afforestation	*R*	Area/Afforestated area	*r*

**Note**: The relative annual rates of change (%) of the six driving factors in the revised IPAT equation (i.e., *p*, *a*, *c*, *t*, *s* and *r*) are numerically equal to the corresponding derivatives for each year after taking the common logarithm [please see Eqs (2–8) for more details]. Values of *t* < 0 indicate that the proportion of imported wood in China’s total wood consumption becomes large, indicating that wood orientation policy tends to increase wood imports; values of *t* > 0 indicate that the wood orientation policy mainly relies on the extraction of wood from domestic forests, indicating an increasing intensity of deforestation. Similarly, values of *s* < 0 show that deforestation (i.e., cutting down China’s domestic forests) is stronger than afforestation, suggesting a low level of sustainable management; on the contrary, a high level of sustainable management of forest is currently undergoing. Values of *r* < 0 indicate that the effect on forest area change caused by afforestation is gradually increasing; on the contrary, a decreasing contribution of afforestation to China’s forest area change is currently undergoing.

As described above, the revised IPAT model was written as:

I=P×A×C×T×S×R (2)

Thus,

lg(I)=lg(P)+lg(A)+lg(C)+lg(T)+lg(S)+lg(R) (3)

We assumed that lg(*I*), lg(*P*), lg(*A*), lg(*C*), lg(*T*), lg(*S*) and lg(*R*) have a linear relationship with time, respectively, and then,

dlg(I)/dt=dlg(P)/dt+dlg(A)/dt+dlg(C)/dt+dlg(T)/dt+dlg(S)/dt+dlg(R)/dt (4)

Let *i* ≈ *d*lg(*I*)*/dt*, *p* ≈ *d*lg(*P*)*/dt*, *a* ≈ *d*lg(*A*)*/dt*, *c* ≈ *d*lg(*C*)*/dt*, *t* ≈ *d*lg(*T*)*/dt*, *s* ≈ *d*lg(*S*)*/dt*, *r* ≈ *d*lg(*R*)*/dt*

Then,

i=p+a+c+t+s+r (5)

The definition of each symbol in Eqs (2–5) is as specified in Table 1.

We employed Eqs (6) and (7) to respectively calculate the 24-year absolute change (*slope*) and relative annual rates of change (*RR*, %) of the six driving fators, namely, Chinese population, affluence (i.e., GDP per capita), wood consumption intensity, policy of importing wood, sustainable management level and afforestation impact on a logarithmic basis at the national level over the past 25 years, as follows:

y=slope×x+b (6)

where *y* represents the 24-year population, affluence, consumption intensity, policy of importing wood, sustainable management level or afforestation impact on a logarithmical basis at the national level, *x* is the corresponding specific year, and the *slope* denotes the absolute amplitude and the direction of changing. We calculated the 24-year relative annual rates of change as follows:

$$RR,\%=(slope/\frac{1}{24}\sum_{i=1}^{24}y_{i})\times 100$$ (7)

where *y*_*i*_ denotes the population, affluence, consumption intensity, policy of importing wood, sustainable management level or afforestation impact on a logarithmical basis at the national level over the past 24 years, and the *slope* and *RR* (%) are the corresponding absolute amplitude and 24-year relative annual rates of changes of the six driving factors, respectively.

Let

Sum=|p|+|a|+|c|+|t|+|r|+|s| (8)

Then changes in the forest area can be decomposed into the combination of the six components specified by Eq (8).

Therefore, the contribution weights (*W*, %) (i.e., the weights representing the contributions of the specified factors) can be calculated using Eq (9), as follows:

W,%=W/Sum×100% (9)

where *W* is the contribution weight of the population, affluence, consumption intensity, policy of importing wood, sustainable management level or afforestation impact at the national level, and *Sum* denotes the sum of the absolute values of the driving factors in Eq (8).

To explore the forest area changes with time, we employed Eqs (10) and (11) to calculate the absolute change rate (*AR*) and relative change rate (*RR*) of forest area. The *AR* was defined as the regression coefficient *a* of the interpreted forest area of each time span versus the corresponding time span [please see Eq (10) for more details], while *RR* was the annual relative change rate of forest area, equal to *AR* divided by the mean forest area of the six time spans [i.e., Eq (11)].

y=ax+b (10)

where *y* represents the RS-derived forest area of each time span, and *x* is the corresponding median year of 1982–1983, 1984–1988, 1989–1993, 1994–1998, 1999–2003 and 2004–2006, respectively; *a* and *b* are regression coefficients.

$$RR(\%/yr)=\left[a/\left(y_{1}+y_{2}+y_{3}+y_{4}+y_{5}+y_{6}\right)/6\right]\times 100$$ (11)

where *RR* represents the annual relative change rate of forest area (i.e., *i* in Eq 5), and *y*_1_, *y*_2_, *y*_3_, *y*_4_, y_5_, *y*_6_ and *a* and is RS-derived forest area of time spans of 1982–1983, 1984–1988, 1989–1993, 1994–1998, 1999–2003 and 2004–2006, and regression coefficient *a* in Eq 2 (i.e., *AR*), respectively.

As seen from the aforementioned equations, the annual relative change rate of forest area (i.e., *i*) was calculated from RS-derived NDVI via Eqs (10) and (11), while the rate of the six driving factors (i.e., *p*, *a*, *c*, *t*, *s* and *r*) were respectively calculated using the compilation of data mentioned in Section “Other data” via Eqs (6) and (7).

RS-derived forest areas (NDVI, actually) were estimated at the province level for the time periods of 1982–1983, 1984–1988, 1989–1993, 1994–1998, 1999–2003 and 2004–2006. During the study period, only the four NFIs (i.e., 3^rd^, 4^th^,5^th^ and 6^th^) at the province level were available, and thus forest area derived from NDVI was respectively compared with that of NFI only in the time periods 1984–1988, 1989–1993, 1994–1998 and 1999–2003 to show the discrepancy (or departure) in forest area estimated using RS and NFI.

Root-Mean-Square-Error (*RMSE*) and Relative-Root-Mean-Square-Error (*RRMSE*) were used, as the two indicators are regularly used in the inter-comparison of model performance (e.g., [53, 54]). The comparison in this study was conducted province by province. *RMSE* and *RRMSE* were respectively defined as the following equations:

$$RMSE=\sqrt{\frac{\sum_{i=1}^{30}{\left(I_{i}-NFI_{i}\right)}^{2}}{n}}$$ (12)

$$RRMSE=\frac{RMSE}{NFI_{mean}}\times 100$$ (13)

$$NFI_{mean}=\frac{\sum_{i=1}^{30}NFI_{i}}{30}$$ (14)

where *I*_*i*_ and *NFI*_*i*_ denote the provincial forest area (10^6^ ha) of interpretation and NFI for a certain time span, *i* = 1, 2, 3,⋯ 30, and *NFImean* is the mean of the 30-province NFI forest area (10^6^ ha) of the same time span.

## Results

### Provincial forest area derived from NDVI and its comparison with that of NFI

**Fig 1:**
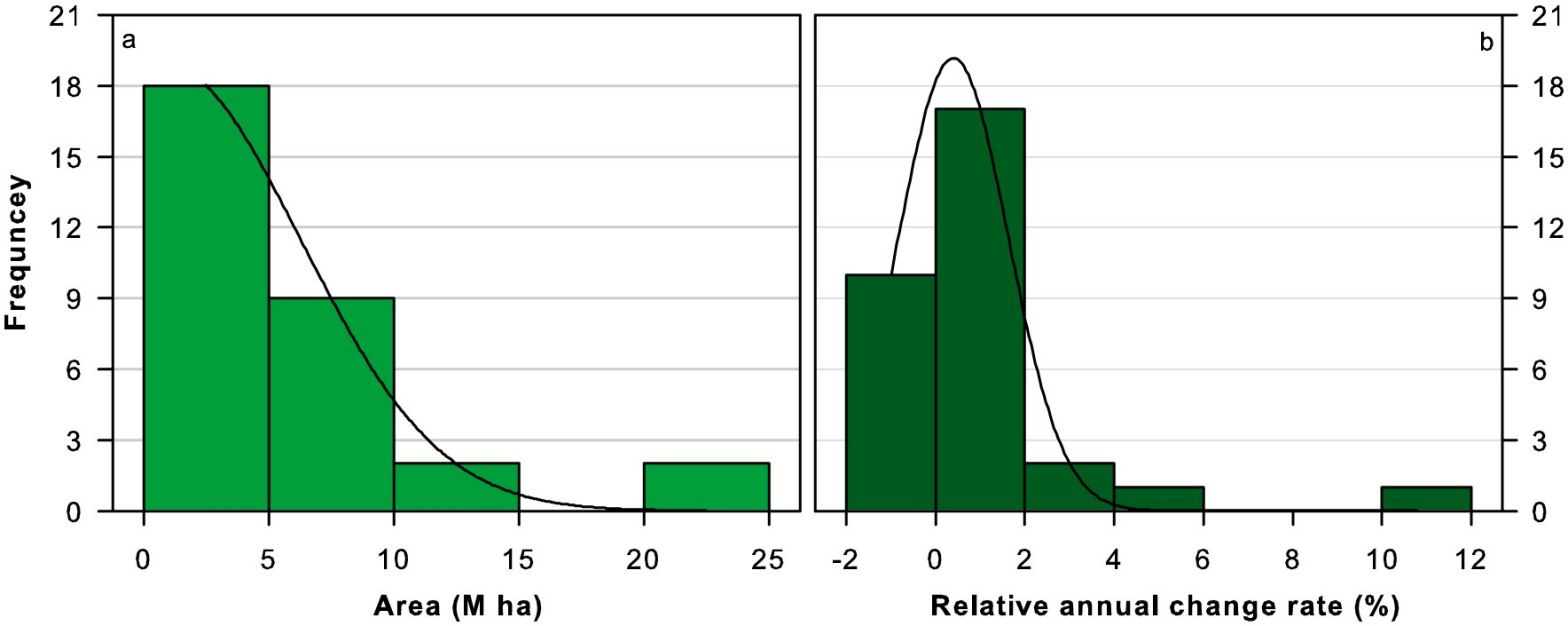
Frequency map of forest areas at the province level (a) and their relative annual change rate (b).

Following the produced thematic map of forest distribution, it was easy to calculate the provincial forest areas for the time spans of 1982–1983, 1984–1988, 1989–1993, 1994–1998, 1999–2003 and 2004–2006, respectively (Table 2). China’s mean forest area during the study period was estimated to be approximately 1.69×10^8^ hm^2^, with a forest coverage of 17.62%. China’s forest area showed a reverse J-shape at the province level (Fig 1a). Among 31 provinces, most (18) was less than 5 million hectares in forest area, 4 were larger than 10 million hectares, and others ranged between the two. Forest area was the largest in Heilongjiang Province (2.07×10^7^ hm^2^), contributing to approximately 12.2% of the total national forest area, while Fujian Province had the largest forest coverage (> 50%) in mainland China.

**Table 2:** NDVI-derived forest areas (10^4^ ha) at the province level for the six time spans and their annual relative change rates.

Province/Municipalities/Autonomous regions	1982–83	1984–88	1989–93	1994–98	99–2003	2004–06	Mean	Forest coverage (%)	RR (%)
Heilongjiang	2110.72	2015.36	2215.04	2094.72	1957.12	2001.92	2065.81	45.44	0.25
Inner Mogolia	1865.6	1960.32	2037.12	2158.72	1941.12	2195.84	2026.45	17.49	0.51
Sichuan	1445.76	1551.36	1520.64	1413.76	1443.2	1612.16	1497.81	26.46	0.14
Yunnan	1246.72	1386.88	1365.76	1473.92	1461.12	1361.28	1382.61	36.13	0.40
Tibet	859.52	992	1036.16	1013.12	955.52	1020.16	979.41	7.97	0.39
Jilin	778.88	725.76	833.28	785.92	755.84	709.12	764.80	40.49	-0.24
Jiangxi	877.44	769.28	821.76	781.44	718.72	567.68	756.05	45.35	-1.39
Guangdong	708.48	709.12	813.44	661.12	695.04	704.64	715.31	40.21	-0.20
Hunan	735.36	653.44	764.16	824.96	627.2	588.16	698.88	32.99	-0.65
Shaanxi	639.36	632.32	677.12	729.6	631.04	706.56	669.33	31.88	0.35
Guangxi	668.16	611.84	746.24	704.64	627.84	513.92	645.44	27.16	-0.72
Fujian	707.84	609.28	726.4	610.56	533.12	514.56	616.96	50.78	-1.30
Zhejiang	441.6	513.28	537.6	531.2	518.4	460.16	500.37	49.15	0.11
Liaoning	428.16	461.44	501.76	483.2	460.16	544	479.79	32.92	0.69
Hubei	418.56	498.56	378.88	436.48	418.56	453.12	434.03	23.35	-0.04
Guizhou	283.52	312.32	380.16	344.32	336	468.48	354.13	20.07	1.63
Hebei	267.52	300.8	314.88	315.52	266.88	370.56	306.03	16.46	0.79
Henan	250.88	238.08	270.72	297.6	252.8	293.12	267.20	16.00	0.65
Xinjiang	237.44	223.36	209.28	309.76	313.6	281.6	262.51	1.59	1.43
Gansu	258.56	280.96	238.72	243.2	272.64	238.72	255.47	5.68	-0.29
Anhui	179.84	234.24	234.24	273.28	278.4	261.76	243.63	17.63	1.45
Qinghai	134.4	226.56	232.96	215.68	193.28	233.6	206.08	2.86	1.04
Taiwan	187.52	195.84	207.36	210.56	195.84	202.24	199.89	55.90	0.23
Shanxi	191.36	185.6	194.56	210.56	169.6	209.92	193.60	12.36	0.18
Shandong	134.4	167.04	230.4	227.84	172.8	164.48	182.83	12.01	0.52
Hainan	102.4	136.96	145.28	121.6	128	114.56	124.80	36.59	0.01
Jiangsu	30.08	24.96	30.08	54.4	44.8	43.52	37.97	3.70	2.50
Beijing	10.24	8.96	26.88	29.44	16.64	48	23.36	13.11	5.57
Ningxia	14.08	13.44	14.08	13.44	16	25.6	16.11	2.43	2.42
Tianjing	9.6	8.96	7.68	8.32	7.04	8.96	8.43	7.33	-0.59
Shanghai	0	0.64	0.64	2.56	5.76	6.4	2.67	4.48	11.63
Total	16224	16649	17713	17581	16414	16925	16918	17.61	0.089

**Fig 2:**
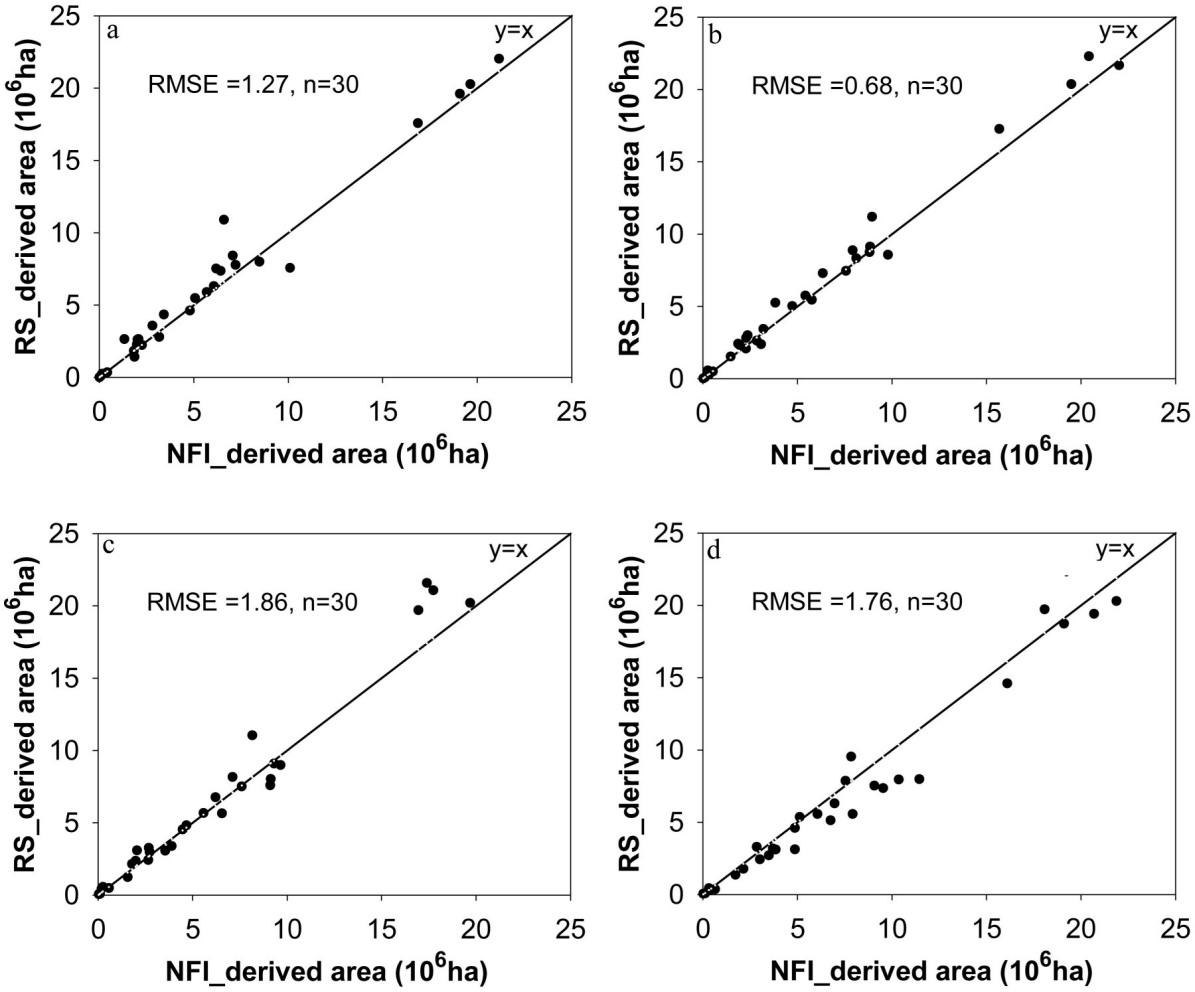
Comparison in forest area (10^6^ ha) based on remote sensing technique (RS) and NFI data in the time periods of 1984–1988 (a), 1989–1993 (b), 1994–1998 (c) and 1999–2003 (d), respectively. Each point denotes a province.

NDVI-derived forest area was compared to that reported in NFI. Four NFIs were reported during the study period of 1982–2006, namely, the 3^rd^ (1984–1988), 4^th^ (1989–1993), 5^th^ (1994–1998) and 6^th^ (1999–2003) NFIs. The comparisons showed that all estimated provincial forest areas (points in Fig 2) were near the 1:1 line with respective RMSEs less than 2, suggesting that NDVI-derived forest areas were all consistent with that derived from NFI of the same time span.

### Change in China’s forest area

**Fig 3:**
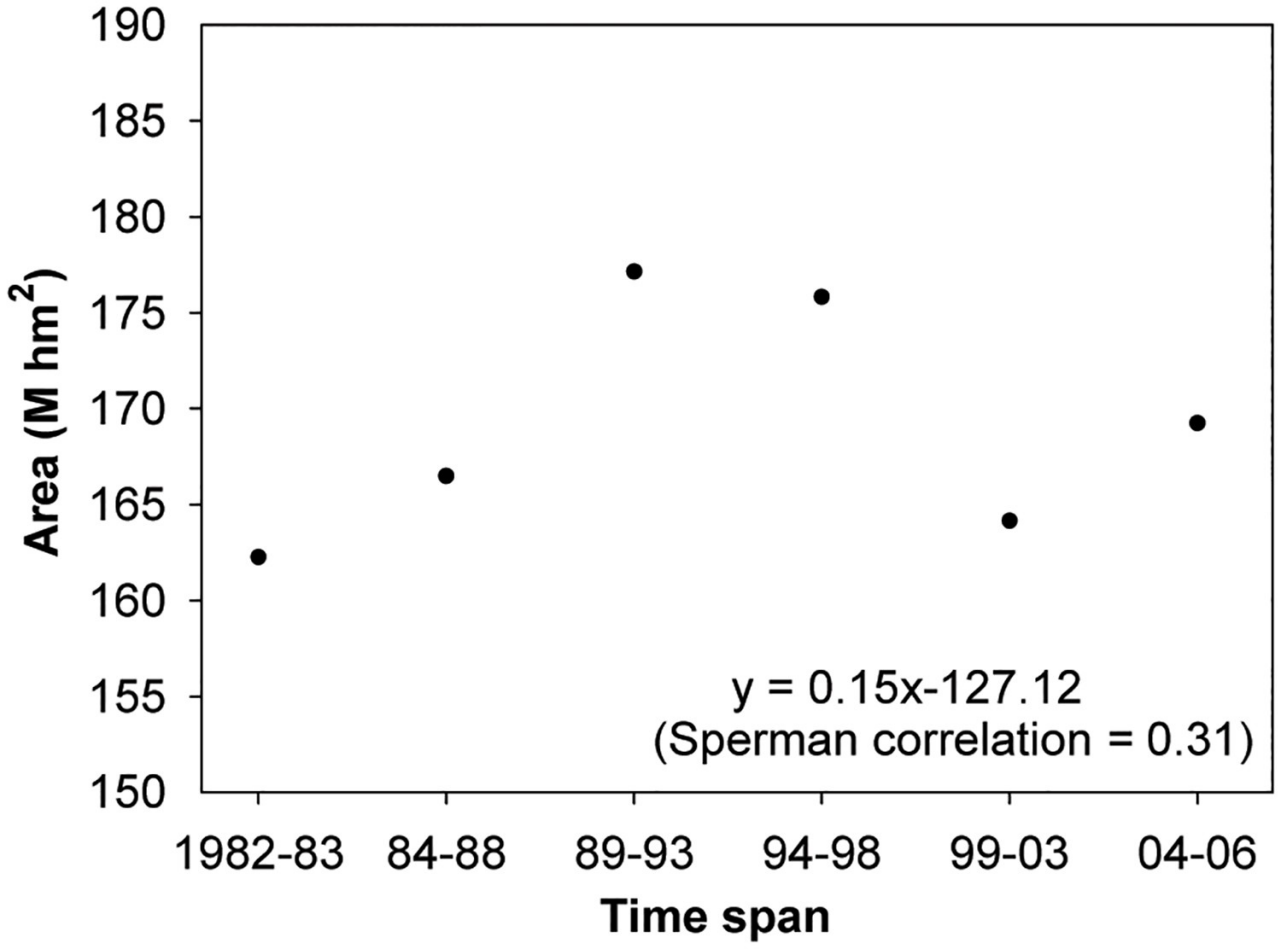
Scatter plot showing the forest area for the six time spans from 1982 to 2006.

Overall, China’s forest area increased with a fluctuation (Fig 3). The annual absolute increase or increment (i.e., *AR*) in national forest area was estimated to be approximately 1.5×10^5^ hm^2^, equivalent to 0.089% of the relative annual change (increase) rate.

**Fig 4:**
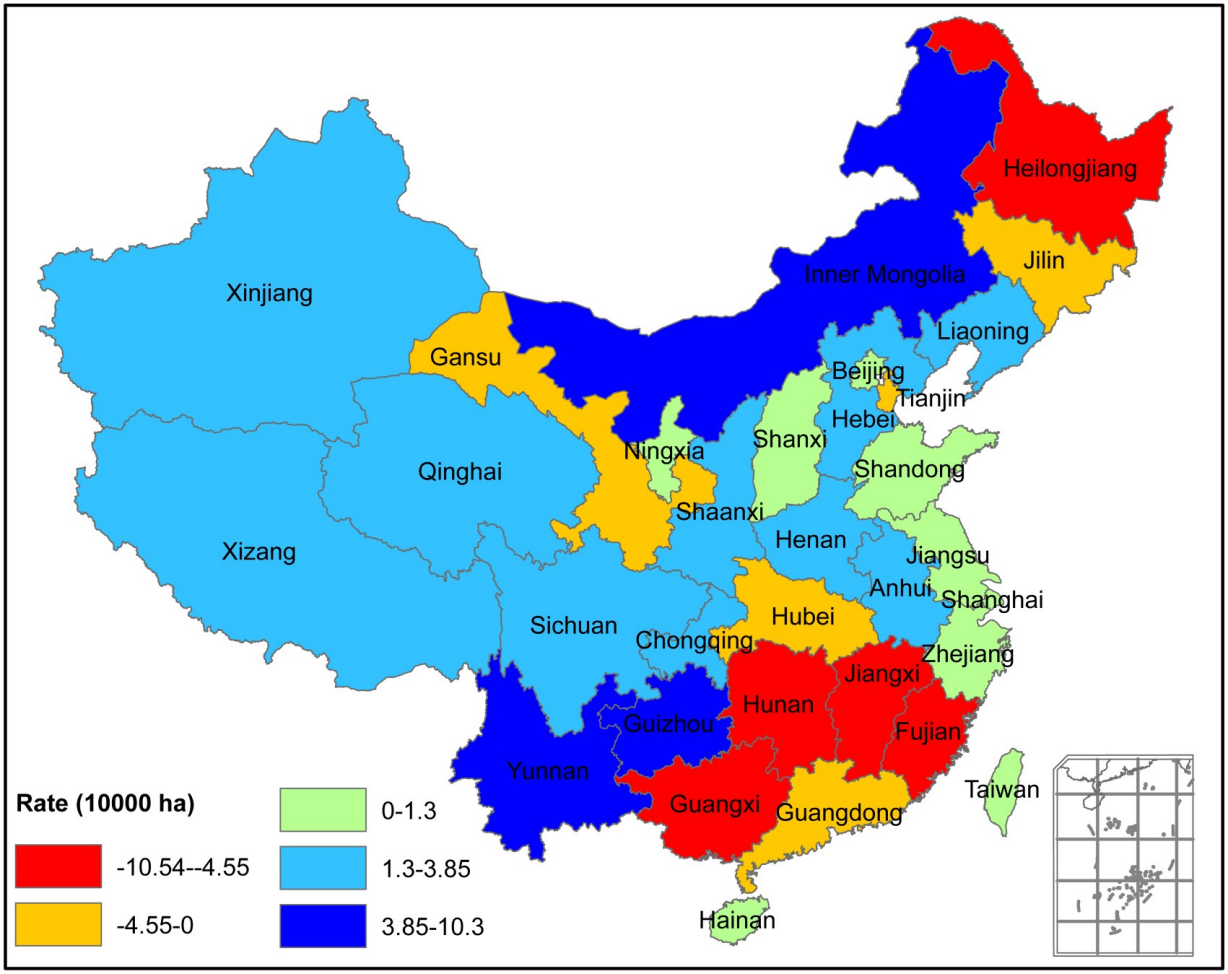
Annual change rate of forest area at the province level over the past 25 years.

At the province level, a large difference in the changing rate and direction of forest area was found (Fig 4). Over the past 25 years, forest area has declined in 10 provinces, mainly in Northeastern and Southern China. Heilongjiang, Jiangxi, Fujian, Hunan and Guangxi Provinces had an annual decreasing rate > 4.55×10^4^ hm^2^. In contrast, 21 provinces showed an increase in forest area with a positive annual change rate ranged from 1.3×10^4^ hm^2^ to 3.85×10^4^ hm^2^; however, a larger annual increasing rate > 3.85×10^5^ hm^2^ occurred in Inner Mongolia, Guizhou and Yunnan Provinces.

Similarly, the relative annual change rate and direction of provincial forest area also differed with province (Fig 1b). Twenty-one provinces had positive relative annual change rates, while 10 had negative ones. The relative annual change rates in 27 out of 31 provinces ranged from -2% to 2%. Shanghai had the largest relative annual change rate (11.62%), followed by Beijing (5.57%), Jiangsu (2.50%) and Ningxia (2.42%), respectively.

**Fig 5:**
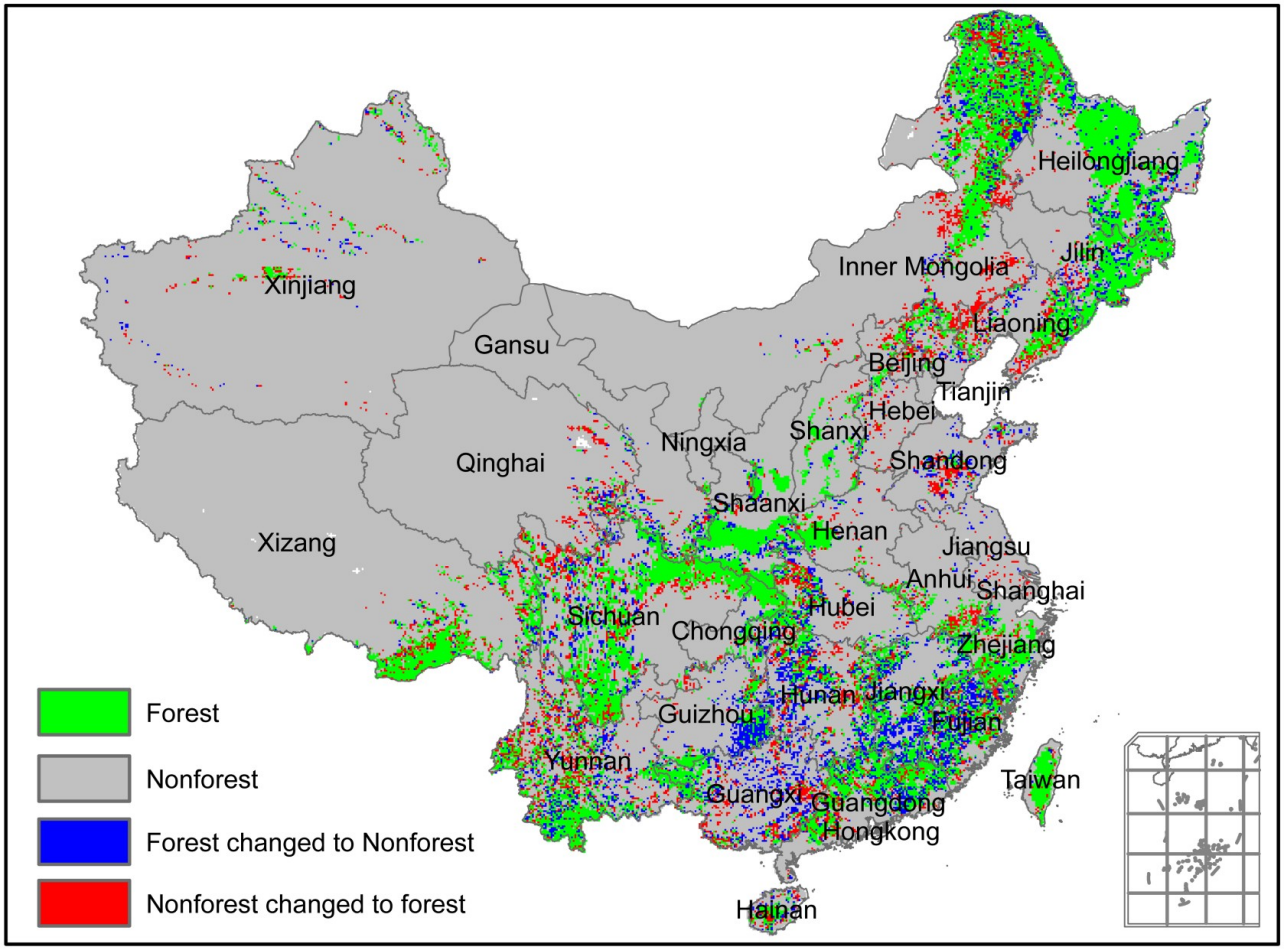
Spatial distribution of change in China’s forest over the past 25 years.

In addition, a transfer matrix was used to analyze mutual transformation between forest and non-forest in the past 25 years (Fig 5). Approximately 860.36 million hectares of land cover type remained unchanged, including 136.62 million hectares of forest and 723.74 million hectares of non-forest, respectively. Additionally, 99.92 million hectares of land cover type was transformed from non-forest to forest or from forest to non-forest, accounting for 10.40% of China’s land area. The transformed area from non-forest to forest, and from forest to non-forest were estimated as 51.76 and 48.16 million hectares, respectively. Thus, the net increment of forest area was 3.60×10^6^ hm^2^ (3.60×10^6^ = 51.76×10^6^−48.16×10^6^), accounting for 2.13% of China’s mean forest area [2.13% = (3.60 ×10^6^)/(169.18×10^6^)×100%].

### Influential factors driving the change in forest area

Based on Eqs (2–9) described above and the compiled data of the Chinese population (logarithmic *P*), affluence (logarithmic *A*), wood consumption intensity (logarithmic *C*), policy of importing wood (logarithmic *T*), sustainable management level (logarithmic *S*) and afforestation impact (logarithmic *R*) for each time period (Part I in Table 3), it was not difficult to calculate the change rate (i.e., *slope*), relative annual change rate (i.e., *RR*) and contribution weights (i.e., *W*) of the possible driving factors (Part II in Table 3).

**Table 3:** Relative change rates and contribution weights of each driving factor in the revised IPAT model.

Part I						
Time span	lg(*P*)	lg(*A*)	lg(*C*)	lg(*T*)	lg(*S*)	lg(*R*)
1982–1983	8.9954	1.8918	-3.1310	-0.0455	-0.9775	1.4770
1984–1988	9.0140	2.1346	-3.2912	-0.0535	-0.9647	1.3821
1989–1993	9.0558	2.4477	-3.6977	-0.0313	-1.0300	1.5038
1994–1998	9.0835	2.8743	-4.1216	-0.0239	-1.1126	1.5454
1999–2003	9.1024	2.0838	-4.3700	-0.1403	-0.8763	1.4156
2004–2006	9.1104	3.3319	-4.5019	-0.1777	-1.1234	1.5892
Part II						
Change	*d*lg(*P*)/*dt*	*d*lg(*A*)/*dt*	*d*lg(*C*)/*dt*	*d*lg(*T*)/*dt*	*d*lg(*S*)/*dt*	*d*lg(*R*)/*dt*
*slope*	0.0054	0.0645	-0.0649	-0.0057	-0.0032	0.0043
*RR*,%	0.0590	2.4534	1.6847	7.1790	0.3136	0.2922
*W*,%	0.4928	20.4761	14.0606	59.9151	2.6171	2.4383

It was concluded from the change rate that the Chinese population, affluence (i.e., GPD per capita) and the ratio of afforestation area to China’s total forest area increased from 1982 to 2006. A negative wood consumption per unit GDP (-0.0649) indicated an increasing efficiency in wood utilization. The negative logarithmic *S* and *T* indicated that China has had a low level of sustainable forest management but a high-degree of dependence on wood imports over the past 25 years.

As for contribution weights, the logarithmic *T* is the largest (*c*.*a*., 59.92%), indicating that the timber import and export policy is likely to play a key role in forest area change in China. Affluence (logarithmic *A*) is the second most important influential factor (*c*.*a*., 20.48%), indicating that it was a secondary factor driving the change of forest area in China. A combination of the two contributed more than 80% (80.4% = 20.48%+59.92%) of the total contribution of the six factors of the IPAT model. Wood consumption intensity contributed to more than 10% (*c*.*a*., 14.06%) of the total. In contrast, the sustainable management level and afforestation effect both made little contribution (< 3%). The Chinese population had almost no effect on China’s forest area change.

## Discussion

Based on the classified forest distribution thematic map derived from NOAA/AVHRR NDVI datasets, China’s forest area was estimated to have an average of 169.18 million hectares with a forest coverage of 17.62% (Table 2). Over the past 25 years, the transformed area from non-forest to forest, and from forest to non forest were estimated as 51.76 and 48.16 million hectares, respectively (Fig 5). Thus, the net increment in forest area was 3.60 million hectares with an annual increase of approximately 0.15 million hectares per year (Fig 3), equivalent to 0.089% of the relative annual change rate (Table 2).

A large difference in the changing rate and direction of forest area at the province level was found (Fig 4). From 1982 to 2006, forest area has declined in 10 provinces, mainly in Northeastern and Southern China, while 21 provinces showed an increase. Similarly, the relative annual change rate and direction of provincial forest area also differed with province. NDVI-derived forest areas were consistent with those reported in 3^rd^, 4^th^, 5^th^ and 6^th^ NFIs, respectively (Fig 2). As far as the changing trend of forest area is concerned, the estimated areas from RS and NFI were generally the same; both showed an upward trend (Fig 3). NFI had a monotonous increase in area, but RS-area showed no obvious increase in the fluctuations. This difference in amplitude is most likely caused by the rationales and estimation errors of the two estimation methods. This study’s results suggested that RS is a very useful and reliable tool to examine large-scale forest area, despite previous studies having noted that estimated forest areas based on RS and NFI data were inconsistent [55].

The revised IPAT model has revealed that the policy of wood import and export is the chief cause in forest area change in China. The forestry policy in China is strongly regulated by Chinese government. From 1982 to 1994, the reform and opening up began, and special funds were designated towards the import of wood, changing the former “self-sufficiency” approach to wood supply. Compared to the time period of 1982–1983, the forestry policy was oriented towards an increase in the quantity of wood imports over the past 25 years. The change in forestry policy thus alleviated the pressure on the destruction of domestic forests and temporarily protected domestic forest resources. A number of studies have shown that importing more wood benefits the importing countries but transfers the ecological consequences of deforestation to exporting countries, as more forests must be destroyed to produce wood that is exported from the exporting countries (often from undeveloped regions and countries) [56–58]. It is possible that the beneficiaries (i.e., the countries importing wood) can thereby alleviate the pressure on the destruction of their own forests and thus temporarily protect their domestic forest resources. China has become the world’s second-largest wood-consumer country after the U.S., and total imports reached 29.57 million m^3^ in 2008 [59]. It is thus not surprising that the policy of wood import and export plays a major role in driving forest area change.

**Fig 6:**
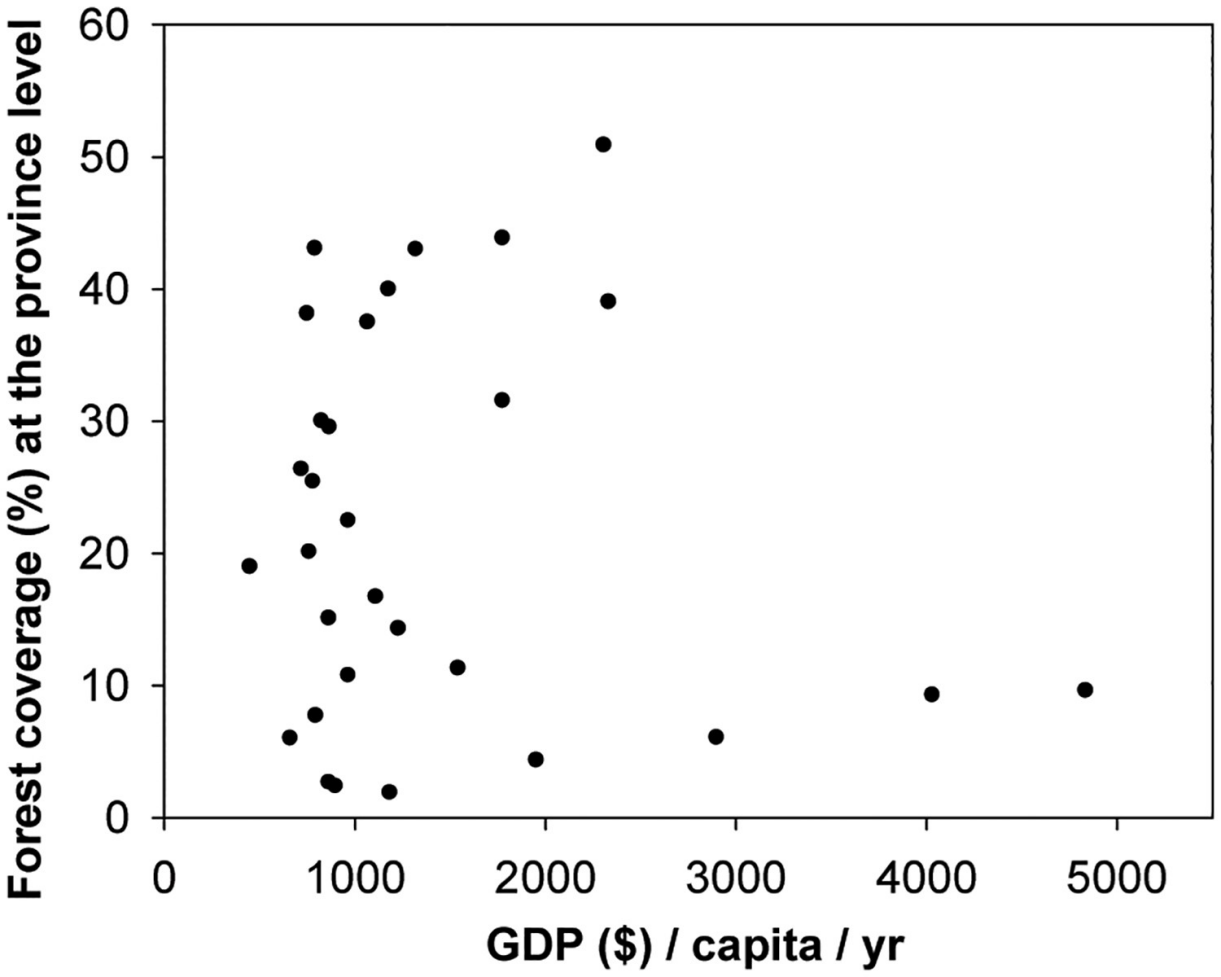
Relationship between forest coverage (%) derived from remote sensing data and the corresponding per capita gross domestic product at the provincial level in China.

Affluence is the second most important influential factor in forest area change. Recent reports have proven that GDP or income per capita has a strong effect on forest cover change [60, 61]. The relationship is known as the environmental Kuznets curve (EKC), which is the inverted U-shaped relationship between income growth and deforestation. However, the relationship between provincial forest coverage and per capita GDP in this study does not agree with the EKC (Fig 6).

It is also noted that wood consumption intensity contributed to more than 10% (*c*.*a*., 14.06%) of the total. Although an increasing efficiency in wood utilization has occurred from 1982 to 2006 (-0.0649 in Table 3), it will probably play a more important role in driving forest cover change in the future, taking China’s recent generous investment and talent incentives into account. As noted in forest transition theory, technological innovation could change or influence the process or direction of forest transition [62, 63]. At present, China’s comprehensive timber utilization rate is only 60%, far lower than the 80% (or even higher) of developed countries; if increased to 80%, then 33% of the current total wood consumption would be saved, equivalent to cutting down approximately 1.655 million hectares less of forest [64].

Some studies have suggested that on different scales the population has a substantial impact on the domestic forest resource. For example, the annual growth of 3% in the deforestation in Africa is consistent with population growth during the same period. Moreover, wood removals are gradually increasing globally with the long-term increases in populations and incomes, and this trend will continue in the coming decades [48, 65]. However, this study does not support this conclusion. Compared with the forestry policy on wood import and export, level of economic development, and the intensity of wood consumption, population has almost no effects on the changes in China’s forest area. Therefore, it is highly probable that the effect of demographic factors on the national forest area will show considerable regional differences.

In this paper, we revised the IPAT model to explore the factors driving China’s forest area. We have only examined the aforementioned six factors, but they may be inadequate. It has been reported that topographical factors such as slope and altitude are also possible influential factors [66].

## Conclusions

Overall, NDVI-derived forest area and its change were consistent with that of NFI. China’s forest area was estimated to have an average of 169.18 million hectares with an annual increase of 0.15 million hectares. However, a large difference in the change rate and direction of forest area at the province level was found. The results were most likely attributed to the policy regarding the import and export of timber and affluence (per capita gross domestic product), and both contributed more than 80% of the total contribution of the six factors of the revised IPAT model.

## Supporting information

S1 Fig Main forest types in China and their distributions based on the Vegetation Map of China. Owing to the similarity in NDVI profiles of the same life type in one climatic zone, China’s vegetation types were grouped into 15 types, namely, 1. Cold Temperature and temperature deciduous coniferous forest, 2. Cold temperature and temperature evergreen coniferous forest, 3. Temperate evergreen coniferous forest, 4. Subtropical and tropical evergreen coniferous forest, 5. Temperature evergreen coniferous and deciduous broadleaved mixed forest, 6. Subtropical evergreen coniferous and evergreen broadleaved mixed forest, 7. Temperature deciduous broadleaved forest, 8. Subtropical deciduous broadleaved forest, 9. Subtropical evergreen broadleaved and deciduous broadleaved mixed forest, 10. Subtropical evergreen broadleaved forest, 11. Tropical rainforest and seasonal rainforest, 12. Subtropical and tropical deciduous coniferous forest, 13. Shrub, 14. Bamboo forest, and 15. Other vegetation types.
(TIF)

S2 Fig Land cover types in the Sixth National Forest Inventory Distribution Map (1999–2003). (TIF)

S3 Fig Interpreted thematic maps of China’s main land cover types of the time period of 1984–1988. Labels 1–15 denote the same vegetation types as described in S1 Fig, and Label 16 represents the non-vegetation type.
(TIF)

S4 Fig Interpreted thematic maps of China’s main land cover types of the time period of 1989–1993. Labels 1–16 denote the same land cover types as described in S3 Fig.
(TIF)

S5 Fig Interpreted thematic maps of China’s main land cover types of the time period of 1994–1998. Labels 1–16 denote the same land cover types as described in S3 Fig.
(TIF)

S6 Fig Interpreted thematic map of China’s main land cover types of the time period of 1999–2003. Labels 1–16 denote the same land cover types as described in S3 Fig.
(TIF)

S1 Table Subdivision of China’s forest types based on the Vegetation Map of China by life type and climatic zone. (DOC)

S2 Table Subdivision of China’s forest types based on IGBP 2001 by life type and climatic zone. (DOC)

S3 Table Accuracy assessment of interpreted forest types of the time period of 1999–2003. (DOC)
