# Supplementary figures and images for: NDVI-derived forest area change and its driving factors in China
Source: PLoS One. 2018 Oct 17;13(10):e0205885. doi: 10.1371/journal.pone.0205885 (PMC6192655; doi:10.1371/journal.pone.0205885)

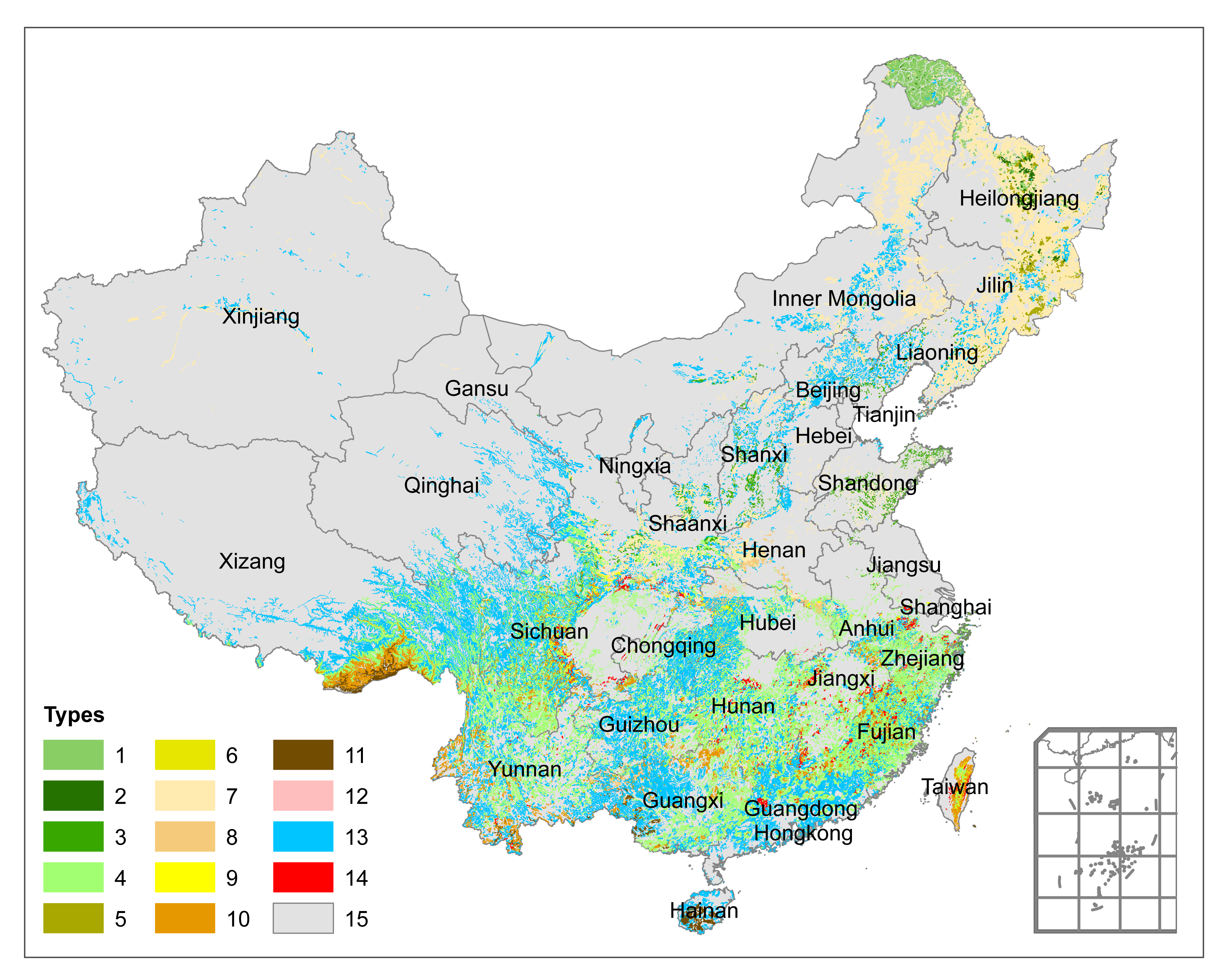

Supplement: S1 Fig — Owing to the similarity in NDVI profiles of the same life type in one climatic zone, China’s vegetation types were grouped into 15 types, namely, 1. Cold Temperature and temperature deciduous coniferous forest, 2. Cold temperature and temperature evergreen coniferous forest, 3. Temperate evergreen coniferous forest, 4. Subtropical and tropical evergreen coniferous forest, 5. Temperature evergreen coniferous and deciduous broadleaved mixed forest, 6. Subtropical evergreen coniferous and evergreen broadleaved mixed forest, 7. Temperature deciduous broadleaved forest, 8. Subtropical deciduous broadleaved forest, 9. Subtropical evergreen broadleaved and deciduous broadleaved mixed forest, 10. Subtropical evergreen broadleaved forest, 11. Tropical rainforest and seasonal rainforest, 12. Subtropical and tropical deciduous coniferous forest, 13. Shrub, 14. Bamboo forest, and 15. Other vegetation types. (TIF) [file pone.0205885.s001.tif]

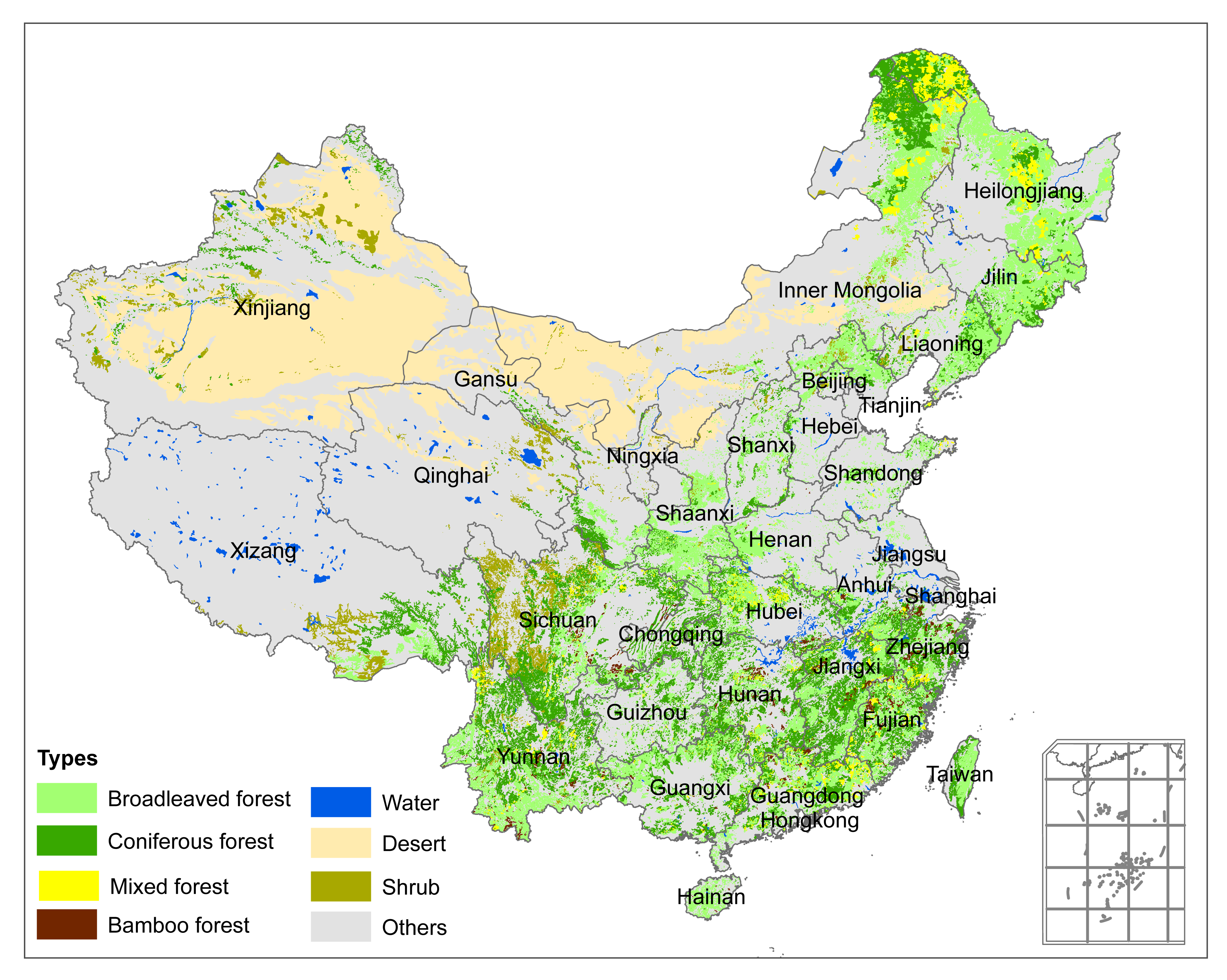

Supplement: S2 Fig — (TIF) [file pone.0205885.s002.tif]

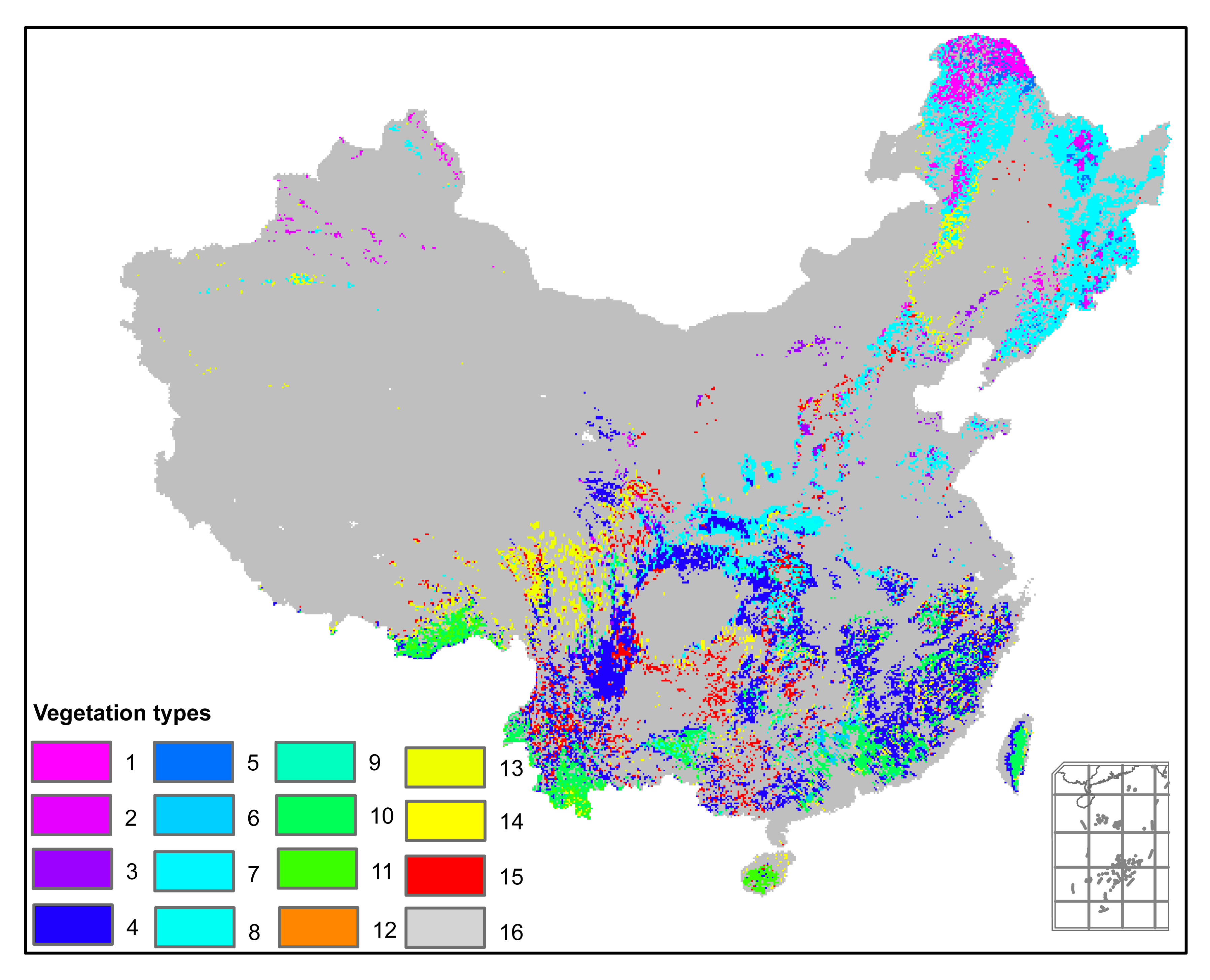

Supplement: S3 Fig — Labels 1–15 denote the same vegetation types as described in S1 Fig, and Label 16 represents the non-vegetation type. (TIF) [file pone.0205885.s003.tif]

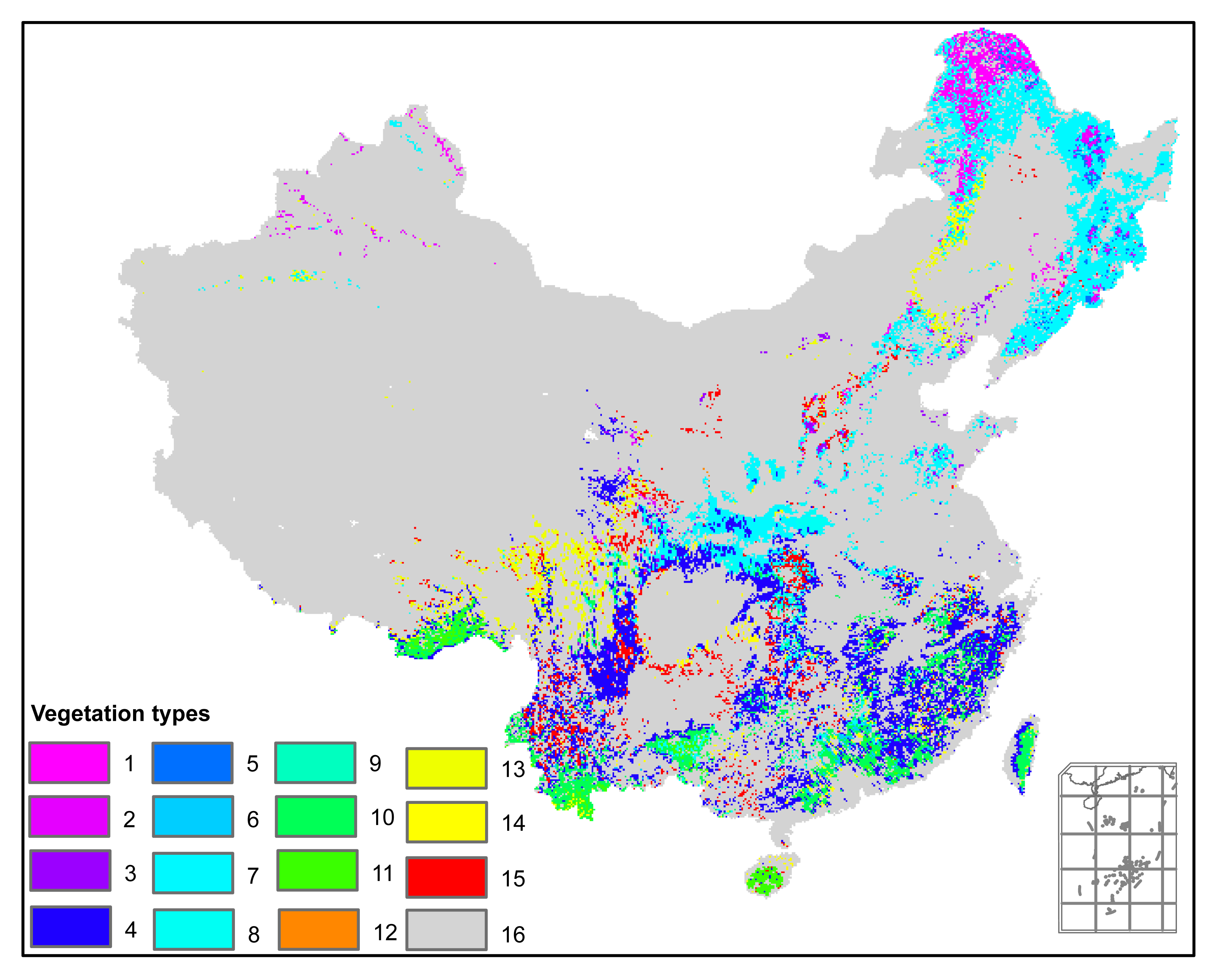

Supplement: S4 Fig — Labels 1–16 denote the same land cover types as described in S3 Fig. (TIF) [file pone.0205885.s004.tif]

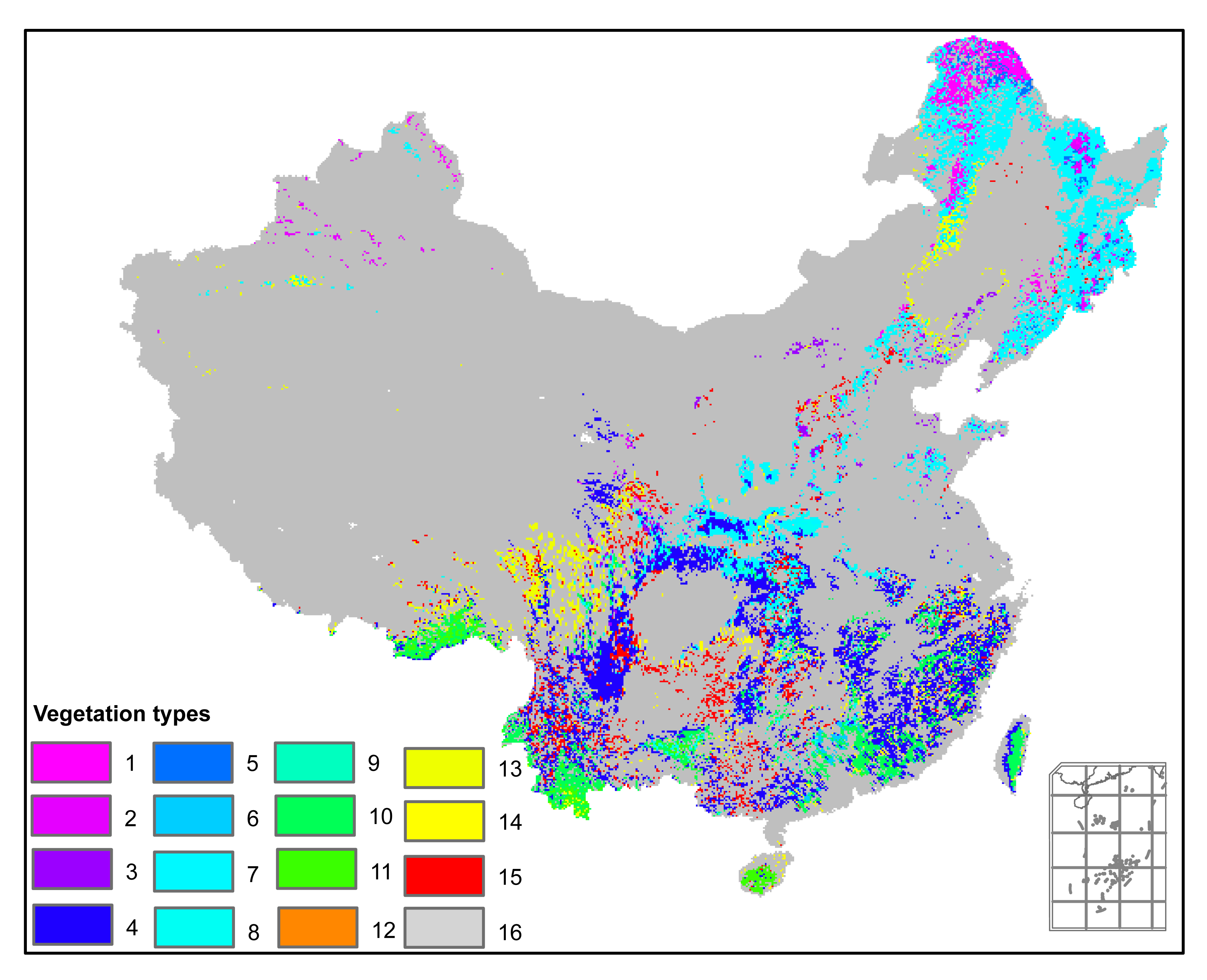

Supplement: S5 Fig — Labels 1–16 denote the same land cover types as described in S3 Fig. (TIF) [file pone.0205885.s005.tif]

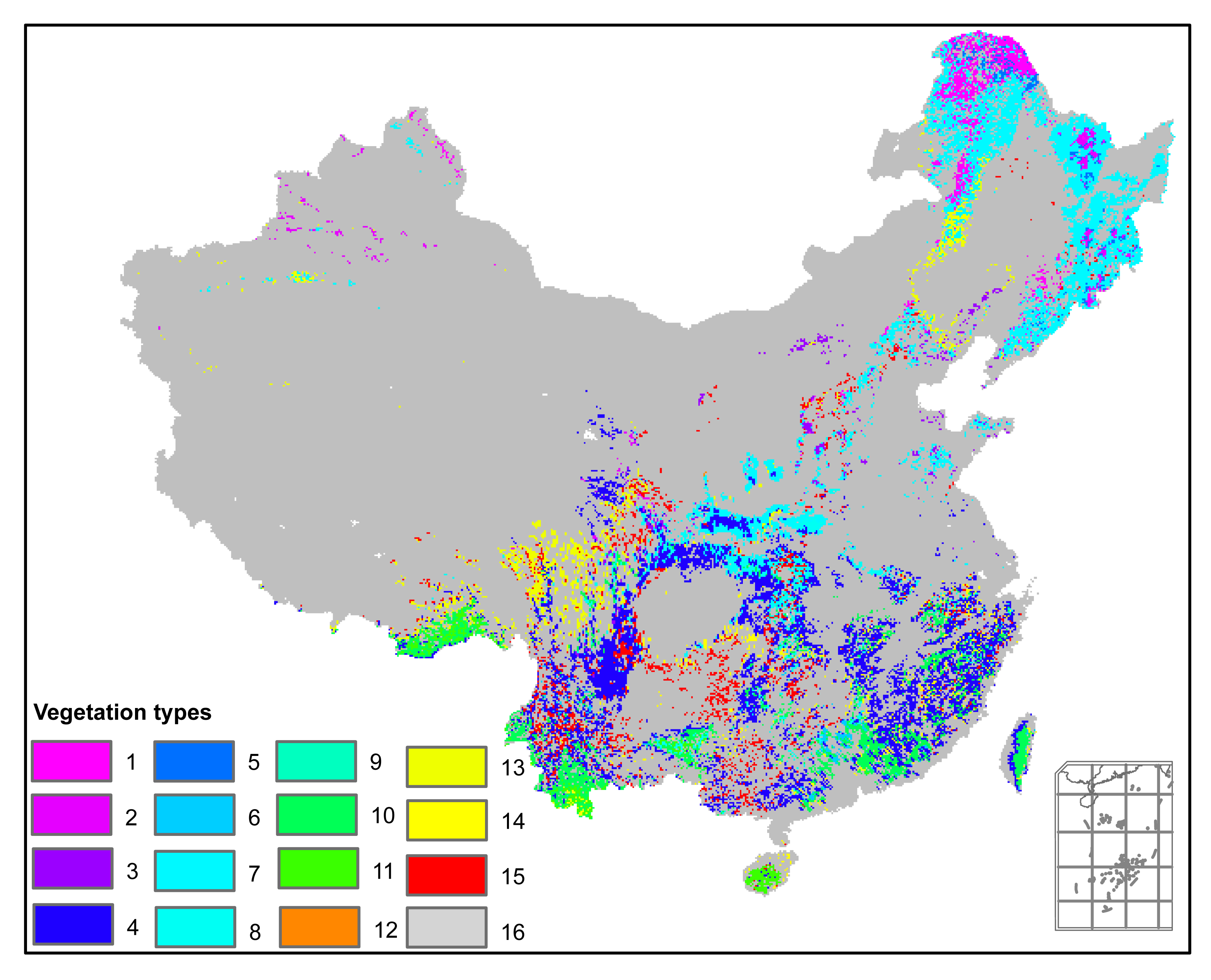

Supplement: S6 Fig — Labels 1–16 denote the same land cover types as described in S3 Fig. (TIF) [file pone.0205885.s006.tif]
