# Supplementary material for: NDVI-derived forest area change and its driving factors in China
Source: PLoS One. 2018 Oct 17;13(10):e0205885. doi: 10.1371/journal.pone.0205885 (PMC6192655; doi:10.1371/journal.pone.0205885)
Supplement: S1 Table — (DOC) [file pone.0205885.s007.doc]

**Table S1 Subdivision of China’s forest types based on Vegetation Map of China by life type and climatic zone**

| **Number** | **Climatic zone** | **Life types** | **Forest types** |  |
| --- | --- | --- | --- | --- |
| 1 | Cold temperate, Temperate | Deciduous, Coniferous | Cold temperature and temperature deciduous coniferous forest |  |
| 2 |  | Evergreen, Coniferous | Cold temperature and temperature evergreen coniferous forest |  |
| 3 | Temperate | Evergreen, Coniferous | Temperature evergreen coniferous forest |  |
| 4 | Tropic, Subtropic |  | Tropic and subtropic evergreen coniferous forest |  |
| 5 | Temperate | Evergreen, Coniferous  Deciduous, Broadleaved | Temperature evergreen coniferous and deciduous broadleaved mixed forest |  |
| 6 | Subtropic | Evergreen, Coniferous  Evergreen, Broadleaved | Subtropic evergreen coniferous and evergreen broadleaved mixed forest |  |
| 7 | Temperate | Deciduou , Broadleaved | Temperature deciduous broadleaved forest |  |
| 8 | Subtropic |  | Subtropic deciduous broadleaved forest |  |
| 9 | Subtropic | Evergreen, Broadleaved  Deciduous, Broadleaved | Subtropic evergreen broadleaved and deciduous broadleaved mixed forest |  |
| 10 | Subtropic | Evergreen, Broadleaved | Subtropic evergreen broadleaved forest |  |
| 11 | Tropic |  | Tropic rainforest |  |
| 12 | Tropic, Subtropic | Deciduous, Coniferous | Tropic and subtropic deciduous coniferous forest |  |
| 13 | Shrub | Shrub | Shrub |  |
| 14 | Bamboo forest | Bamboo | Bamboo forest |  |
| 15 | Other vegetations | - | Other vegetation |  |
| 16 | Non vegetation | - | Non-vegetation |  |
