# Supplementary material for: NDVI-derived forest area change and its driving factors in China
Source: PLoS One. 2018 Oct 17;13(10):e0205885. doi: 10.1371/journal.pone.0205885 (PMC6192655; doi:10.1371/journal.pone.0205885)
Supplement: S2 Table — (DOC) [file pone.0205885.s008.doc]

**Table S2 Subdivision of China’s forest types based on IGBP 2001 by life type and climatic zone**

| **No. IGBP types** | **IGBP 2001** | **Subdivided IGBP 2001** |
| --- | --- | --- |
| 1 | Evergreen Needleleaf forest | Cold temperature and temperature evergreen coniferous forest |
|  |  | Temperature evergreen coniferous forest |
|  |  | Tropic and subtropic evergreen coniferous forest |
| 2 | Evergreen Broadleaf forest | Subtropic evergreen broadleaved forest |
|  |  | Tropic rainforest |
|  |  | Bamboo forest |
| 3 | Deciduous Needleleaf forest | Cold temperature and temperature deciduous coniferous forest |
|  |  | Tropic and subtropic deciduous coniferous forest |
| 4 | Deciduous Broadleaf forest | Temperature deciduous broadleaved forest |
|  |  | Subtropic deciduous broadleaved forest |
| 5 | Mixed forest | Temperature evergreen coniferous and deciduous broadleaved mixed forest |
|  |  | Subtropic evergreen coniferous and evergreen broadleaved mixed forest |
|  |  | Subtropic evergreen broadleaved and deciduous broadleaved mixed forest |
| 6 | Closed shrublands | Shrub |
| 7 | Open shrublands |  |
| 8 | Woody savannas | Other vegetation |
| 9 | Savannas |  |
| 10 | Grasslands |  |
| 12 | Croplands |  |
| 14 | Cropland/Natural vegetation mosaic |  |
| 16 | Barren or sparsely vegetated |  |
| 11 | Permanent wetlands | Non-vegetation |
| 13 | Urban and built-up |  |
| 15 | Snow and ice |  |
