# Supplementary material for: NDVI-derived forest area change and its driving factors in China
Source: PLoS One. 2018 Oct 17;13(10):e0205885. doi: 10.1371/journal.pone.0205885 (PMC6192655; doi:10.1371/journal.pone.0205885)
Supplement: S3 Table — (DOC) [file pone.0205885.s009.doc]

**Table S3 Accuracy assessment of interpreted forest types** of the time period of 1999-2003

| Subdivided China’s forest types of the present paper | Random points | Correct points | Producer’s accuracy | User’s accuracy |
| --- | --- | --- | --- | --- |
| 1. Cold temperature and temperature deciduous coniferous forest | 180 | 161 | 100.00% | 89.44% |
| 2. Cold temperature and temperature evergreen coniferous forest | 45 | 41 | 100.00% | 91.11% |
| 3. Temperature evergreen coniferous forest | 50 | 33 | 100.00% | 66.00% |
| 4. Tropic and subtropic evergreen coniferous forest | 256 | 208 | 99.52% | 81.25% |
| 5. Temperature evergreen coniferous and deciduous broadleaved mixed forest | 20 | 18 | 85.71% | 90.00% |
| 6. Subtropic evergreen coniferous and evergreen broadleaved mixed forest | 30 | 24 | 72.73% | 80.00% |
| 7. Temperature deciduous broadleaved forest | 100 | 80 | 97.56% | 80.00% |
| 8. Subtropic deciduous broadleaved forest | 100 | 92 | 97.87% | 92.00% |
| 9. Subtropic evergreen broadleaved and deciduous broadleaved mixed forest | 20 | 20 | 68.97% | 100.00% |
| 10. Subtropic evergreen broadleaved forest | 100 | 83 | 86.46% | 83.00% |
| 11. Tropic rainforest | 50 | 42 | 100.00% | 84.00% |
| 12. Tropic and subtropic deciduous coniferous forest | 5 | 5 | 100.00% | 100.00% |
| 13. Shrub | 100 | 71 | 49.31% | 71.00% |
| 14. Bamboo forest | 50 | 46 | 100.00% | 92.00% |
| 15. Other vegetation | 100 | 92 | 54.12% | 92.00% |
| Overall accuracy | 84.18% | | | Kappa coefficient = 0.82 |
